# Gender‐dependent association between exhaled nitric oxide and the CC16 38AA genotype in young school children

**DOI:** 10.1002/iid3.332

**Published:** 2020-08-06

**Authors:** Sarah J. D. Nauwelaerts, Nancy H. C. Roosens, Koen De Cremer, Alfred Bernard, Sigrid C. J. De Keersmaecker

**Affiliations:** ^1^ Transversal activities in Applied Genomics Sciensano Brussels Belgium; ^2^ Louvain Centre for Toxicology and Applied Pharmacology University catholique de Louvain Brussels Belgium; ^3^ Platform Chromatography and Mass Spectrometry Sciensano Brussels Belgium

**Keywords:** allergic sensitization, CC16 protein, FeNO, genotype, noninvasive, urine

## Abstract

**Background:**

Studies that investigated the association between the CC16 A38G polymorphism and the risk of asthma yielded conflicting results. The aim of this study among schoolchildren was to assess the relationships of CC16 A38G polymorphism with aeroallergen sensitization and fractional exhaled nitric oxide (FeNO), two outcomes predicting asthma later in life.

**Methods:**

The study included 139 children (72 boys), median age of 7.7. Information on each child's health, lifestyle, and environment was collected through a questionnaire completed by their parents. CC16 genotypes were determined using urinary DNA. We measured FeNO, the CC16 protein in urine and nasal lavage fluid and aeroallergen‐specific immunoglobulin E in nasal mucosa fluid.

**Results:**

Children with the homozygous mutant CC16 38AA genotype had higher odds of increased FeNO (>30 ppb) compared with their peers with the wild‐type genotype 38GG (OR, 9.85; 95% CI, 2.09‐46.4; *P* = .004). This association was female gender specific (*P* = .002) not being observed in boys (*P* = .40). It was also independent of allergic sensitization, which yet emerged as the strongest predictor of FeNO along with the use of bleach for house cleaning. Children with the CC16 38AA genotype had lower covariates‐adjusted urinary CC16 levels than those with 38GG (median, μg/L, 1.17 vs 2.08, *P* = .02).

**Conclusion:**

Our study suggests that the CC16 38AA allele promotes airway inflammation as measured by FeNO through a gender‐dependent association. Deficient levels of CC16 in the deep lung, measured noninvasively in urine, as a possible proxy for serum CC16, might underlie this promoting effect.

## Graphical abstract

Serum CC16 is a biomarker of airway epithelium integrity. We used noninvasive tests in schoolchildren to study the relationship between the CC16 A38G polymorphism and aeroallergen sensitization and exhaled nitric oxide (FeNO), two outcomes predicting asthma later in life. The results showed that CC16 38AA promotes airway inflammation, especially in girls, as measured by FeNO and that decreased levels of CC16 in the deep lung, measured in urine as proxy for serum CC16 might underlie this promoting effect.

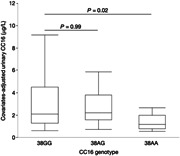

## 1 INTRODUCTION

The club cell protein (hereafter referred to as CC16, formerly known as urine protein 1, uteroglobin, CC10, or Clara cell protein) is a small protein (16 kDa) mainly produced by club cells in the distal bronchioles of the lung, by nasal epithelium cells and all along the trachea‐bronchial tree.^1^, ^2^ In healthy individuals, CC16 secreted in the lining fluid of the respiratory tract, leaks in small amounts across the airway epithelium into the blood from where it is rapidly cleared by glomerular filtration.^2^ The biological function of CC16 has not yet been fully elucidated. However, anti‐inflammatory and immunomodulatory properties^3^, ^4^ including a protective role of CC16 in the respiratory tract are supported by a range of studies associating lower levels of serum CC16 with decreased lung function and increased risks of a number of respiratory diseases.^5^, ^6^, ^7^ Among them is asthma, for which several studies in children have also shown that elevated fractional exhaled nitrogen oxide (FeNO) values are predictive of developing asthma and impaired lung function later in life.^8^

In addition to being a potential biomarker of future lung diseases, serum CC16 is a well‐documented biomarker of the airway epithelium integrity. Serum CC16 rapidly rises when the airway epithelial barrier is disrupted by inflammatory processes or respiratory irritants such as ozone.^9^ When the epithelial barrier is preserved, serum CC16 is a reflection of the number of club cells in the deep lung, which are known to be reduced following chronic exposure to a variety of air pollutants including tobacco smoke and chlorination products in swimming pools.^2^, ^7^ There is also evidence of inherited variations in the CC16 gene expression that, according to some studies, might predispose to respiratory diseases. Indeed, one of the most investigated identified CC16 polymorphisms is characterized by an adenine to a guanine substitution at position 38 (A38G). Some studies have reported associations between this polymorphism and both reduced serum levels of CC16 and an increased incidence of asthma.^10^, ^11^ These findings, however, were not always confirmed.^12^

The measurement of CC16 in serum is not always possible, especially in children, due to ethical reasons. Therefore, noninvasive samples such as urine and nasal lavage fluid (NALF) can be used as alternatives for CC16 measurements.^13^, ^14^ However, an adjustment is required for variations in the epithelial lining fluid (ELF) recovery and for the physiological variations of urine dilution and protein tubular reabsorption capacity in respectively the NALF and urine, to allow correct interpretation of the results. Usually, NALF urea and urinary creatinine are used for this adjustment but the latter only accounts for correcting for variations in dilution in urine and not for tubular reabsorption capacity. Instead, the use of a protein with similar biochemical properties and size as CC16 and which is not influenced by the investigated conditions, would be more suitable as adjuster for protein tubular reabsorption and variations in dilution. The retinol‐binding protein 4 (RBP4) was previously described as a functional biomarker of tubular function.^15^, ^16^ Due to its small size (21.2 kDa) it is rapidly eliminated from plasma by glomerular filtration, then reabsorbed and catabolized in the proximal tubular cells. Levels are expected to be low in healthy individuals, but increase considerably with changing renal tubular function, for instance influenced by exercise or medication intake. Therefore, urinary RBP4 might be considered as a sensitive index of the kidney's ability to reabsorb low molecular weight proteins in the proximal tubuli. However, this protein was not yet investigated as a potential adjuster of variations in dilution and tubular reabsorption capacity when measuring urinary CC16 levels in a population‐based study involving young children.

In the present study among young schoolchildren, we used noninvasive tests to explore the associations of CC16 A38G genotypic status with exhaled nitric oxide (FeNO) and allergic sensitization, both predictors of asthma.^17^ We also examined whether variations in the expression of CC16 linked to the A38G genotypes are reflected by levels of CC16 in urine or NALF.

## 2 MATERIALS AND METHODS

### 2.1 Population

The urine and NALF samples were collected from children recruited in the framework of a 2‐year (2008‐2010) prospective epidemiological study on the impact of environmental stressors on the child's health. Parents completed a questionnaire inquiring about the child's health, lifestyle, and environment. Children participated in the study with the signed informed consent of their parents and their oral assent at the time of examination. The Ethics Committee of the Faculty of Medicine of the Catholic University of UCLouvain approved the study protocol. More details regarding the study set‐up can be found elsewhere.^14^, ^18^

### 2.2 Sample collection and analyses

The protocols for the collection of urine and NALF are described in detail elsewhere.^14^, ^18^ The concentration of nitric oxide (FeNO) was measured in exhaled air with the NIOXTM analyzer (Aerocrine AB, Solna, Sweden) by following the guidelines of the American Thoracic Society. For diagnosing increased FeNO values, we used a cut‐off of 30 ppb, which in our study corresponded to the 95th percentile of baseline values, excluding subjects with asthma or allergic diseases. Aeroallergen sensitization was screened by performing the Rhinostick test, a noninvasive test with similar sensitivity but with a greater specificity than the skin‐prick tests.^19^ The test, screening specific immunoglobulin E (IgE) to cat dander, house dust mite (HDM) and grass, weeds, or tree pollen, was calibrated with serum standards and was categorized as positive at specific IgE ≥ 0.35 kIU/L.^20^ The concentrations of urinary CC16, NALF CC16 and urinary RBP4, selected as potential adjuster for variations in dilution and tubular reabsorption capacity, were measured using immunoassays as described elsewhere.^21^, ^22^ Urea and creatinine were quantified with the Beckman Synchron CX5 Delta Clinical System. To correct for variations in the ELF recovery, we expressed CC16 in NALF as raw concentrations or as concentrations adjusted to the median NALF concentration of urea. The concentrations of CC16 in urine were expressed as raw concentrations or as a ratio to urinary creatinine. Additionally, the urinary CC16 was adjusted for the physiological variations of dilution and protein tubular reabsorption capacity with the geometric mean of RBP4 on the basis of the regression coefficient between the two proteins as obtained by multiple regression analysis (log‐log correlation, *R* ^2^ = .097; *P* < .001). The screening of aeroallergen‐specific IgE and the FeNO test were successfully performed for a total of 139 schoolchildren who were retained for this study. Data for protein quantification in urine and NALF were missing for two subjects while CC16 and urea concentrations in NALF were missing for 9 and 32 subjects, respectively.

### 2.3 DNA extraction from the urine samples and genotyping assay

DNA was extracted from urine from the existing biobank, followed by conducting the genotyping assay for the SNP (single‐nucleotide polymorphism) CC16 A38G polymorphism as previously described.^23^ The CC16 SNP genotyping was successfully performed for the 139 schoolchildren.

### 2.4 Statistical analyses

All continuous variables were described as median with interquartile range (IQR). A log transformation was used for the normalization of FeNO values and of CC16 and RBP4 concentrations. Depending on the type of variable, the differences between boys and girls were compared with the *χ* ^2^ test, the Wilcoxon‐Mann‐Whitney test, the Mann‐Whitney *U* test or Student's *t* test. Differences in CC16 and FeNO levels across CC16 A38G genotypes were assessed by analysis of variance (ANOVA) followed by the Dunett post hoc test. The determinants of FeNO were identified through backward stepwise regression analyses by testing as potential predictors gender, CC16 A38G genotypes, sensitization to HDM, pollen or cat dander, house cleaning with bleach, parental smoking or asthma, breastfeeding, day care attendance, living less than 100 m from a busy street, and lifetime hours of chlorinated pool attendance. The CC16 genotypes and chlorinated pool attendance (tertiles) were tested as dummy variables. The logistic regression models were used to investigate the association between high FeNO values (>30 ppb) and the CC16 38A/G genotypes, while adjusting or stratifying for significant covariates identified by multiple regression analysis. Multiple and logistic regression analyses models were also run by adding an interaction term between gender and the CC16 38A/G genotypes. Trends of higher FeNO odds across the different CC16 A38G genotypes were assessed using the Cochran Armitage test. All analyses were performed using JMP Pro version 14 (SAS Institute Inc, Cary, NC). All *P* values were two‐sided with the level of statistical significance set at *P* < .05.

## 3 RESULTS

The characteristics of the children are described in Table 1, showing no meaningful differences between boys and girls. The cohort included 139 children (72 boys) with a median age of 7.7 years. Thirty‐one children (22.3%) had ever been diagnosed with asthma, hay fever, or perennial allergic rhinitis. Almost all children had ever attended a chlorinated swimming pool and 34 (24.5%) of them were living in a house cleaned with bleach.

**Table 1:** Characteristics of the investigated children (n = 139)

	Boys	Girls	*P* value
Children, N (%)	72 (51.8)	67 (42.8)	.32
Age, y, median (IQR)	7.7 (7.4‐7.9)	7.7 (7.4‐7.9)	.32
BMI, kg/m², median (IQR)	16.3 (15.2‐17.5)	15.9 (15.0‐17.8)	.91
Parental asthma, N (%)	8 (11.1)	8 (11.9)	.88
Smoking during pregnancy, N (%)	10 (13.9)	8.0 (11.9)	.73
Day care attendance, N (%)	35 (48.6)	36 (53.7)	.55
Breastfeeding, N (%)	60 (83.3)	52 (77.6)	.39
Older siblings, N (%)	43 (43)	33 (50)	.21
Parental smoking, N (%)	22 (30.6)	19 (28.4)	.78
Living <100 m from a busy road, N (%)	42 (58.3)	36 (53.7)	.58
House cleaning with chlorine bleach, N (%)	15 (20.8)	19 (28.4)	.30
Indoor and/or outdoor chlorinated pools			
Ever attendance, N (%)	69 (95.8)	64 (95.5)	.93
Lifetime attendance, hours, median (IQR)	231 (87‐406)	224 (78‐406)	.96
Doctor‐diagnosed respiratory diseases, N (%)			
Asthma	7 (9.9)	4 (6.0)	.40
Perennial allergic rhinitis	6 (8.3)	3 (4.6)	.85
Hay fever	7 (9.9)	5 (7.5)	.52

Abbreviation: IQR, interquartile range.

**Figure 1:**
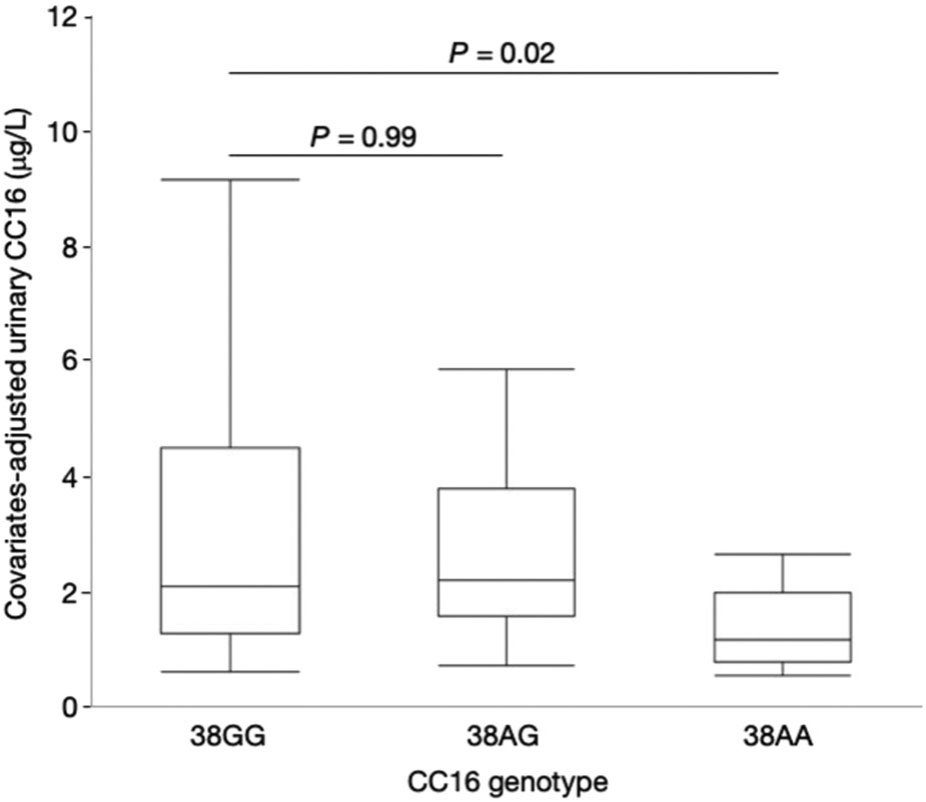
Covariates‐adjusted urinary CC16 according the CC16 A38G genotypes of all the participating children. The middle horizontal bars represent the median values; the upper and lower limits of the boxes, the interquartile range; and the whiskers, the 10th and 90th percentiles. Urinary CC16 level was adjusted for urinary RBP4 and lifetime cumulative attendance at chlorinated pools. *P* values denote the level of statistical significance by the Dunett post hoc test for the comparison with the 38GG genotype

Table 2 shows the results of biomarker measurements, of the CC16 A38G genotyping in children for both sexes and does not show any meaningful differences between both genders. In our study, 25% of children were sensitized to any aeroallergen, 18% to HDM, 17% to pollen, and 6% to cat dander. The prevalence of FeNO > 30 ppb was significantly higher in children with ever asthma (36.8%; *P* = .002) and in those sensitized to aeroallergens (HDM, 24.3%; *P* = .03; pollen, 27.3%; *P* = .047; cat dander, 36.4%; *P* = .02). Results of CC16 A38G genotyping indicate that 51% of the children are homozygous wild‐type 38GG, 38% are heterozygous 38AG, and 11% are homozygous mutant 38AA. The concentrations of CC16 in NALF, crude or adjusted, did not correlate with that in urine, crude or adjusted. There were no significant variations in the crude or urea‐adjusted NALF concentrations of CC16 across the three genotypes. By contrast, as shown in Figure 1, the covariates‐adjusted concentrations of urinary CC16 were significantly lower in children with the mutant 38AA genotype. While the prevalence of doctor‐diagnosed asthma, hay fever, or allergic rhinitis did not vary between the three genotypes, children with the 38AA genotype had significantly higher FeNO levels than those with the 38GG genotype (geometric mean 18.6 vs 10.5 ppb; *P* = .03). This finding was confirmed by the multiple regression analysis showing that the log‐transformed FeNO correlated positively with the mutant 38AA genotype (coefficient, 0.23; 95% confidence interval [95% CI], 0.06 to 0.40; *P* = .008), the use of bleach for house cleaning (coefficient, 0.23; 95% CI, 0.11 to 0.36; *P* <.001) and the sensitization to any aeroallergen (coefficient, 0.10; 95% CI, 0.04 to 0.16; *P* = .002). The model also retained the female gender (coefficient, −0.08; 95% CI −0.18 to −0.024; *P* = .13) and the interaction term between the female gender and the 38AA genotype (coefficient, 0.27; 95% CI −0.07 to 0.61; *P* = .13). On average children living in a house cleaned with bleach had FeNO levels 50% higher and a prevalence of elevated FeNO more than four times higher than those not living in a such a house (geometric mean 15.4 vs 10.1 ppb; *P* = .002; FeNO > 30 ppb; 32.4% vs 7.6%; *P* < .001). Similarly, the sensitization to any aeroallergen, increased the geometric mean of FeNO by 58.5% (15.5 vs 9.77 ppb; *P* = .002) and the prevalence of elevated FeNO by a factor of 2.6 (FeNO > 30 ppb; 22.9% vs 8.8%; *P* = .04). This increased prevalence of elevated FeNO among the children sensitized to any aeroallergen, was more pronounced in boys (40.0% vs 7.0%; *P* = .004) than in girls (26.3% vs 8.5%; *P* = .11). Likewise for the association of FeNO with the CC16 A38G polymorphism, the prevalence of elevated FeNO with house cleaning bleach was significantly increased in children not sensitized to any aeroallergen (FeNO > 30 ppb; 30.0% vs 2.8%; *P* = .001) but not in those sensitized (35.7% vs 17.7%; *P* = .26).

**Table 2:** Allergic sensitization, urinary, and NALF protein biomarker concentrations

	Boys (N = 72^a^)	Girls (N = 67^a^)	*P* value
Aeroallergen‐specific nasal IgE, N (%)			
Any aeroallergen	23 (31.9)	25 (37.3)	.51
House dust mite	18 (25.0)	19 (28.4)	.65
Cat dander	7 (9.7)	5 (7.5)	.64
Pollen	11 (15.3)	12 (17.9)	.68
NALF biomarkers, median (IQR)			
CC16, µg/L	28.7 (13.5‐47.5)	40.8 (19.3‐75.8)	.02
Urea, mg/L	80.5 (63.0‐103)	62.5 (49.3‐89.3)	.048
CC16 adjusted for urea, µg/mg	36.4 (16.5‐55.5)	69.1 (26.9‐131)	.123
Urine biomarkers, median (IQR)			
CC16, µg/L	1.9 (1.7‐2.1)	2.5 (1.6‐4.3)	.28
Creatinine, g/L	0.96 (0.7‐1.3)	0.99 (0.63‐1.18)	.31
RBP4, µg/L	87.1 (57.5‐112)	92.3 (63.4‐128)	.54
CC16, µg/g creatinine	2.1 (1.2‐3.7)	1.4 (0.7‐3.3)	.043
CC16 adjusted to RBP4, µg/L	2.4 (1.5‐4.6)	2.1 (1.3‐4.2)	.26
CC16 SNP A38G genotype, N (%)			
Homozygous wild‐type (CC16 38G/G)	36 (50.0)	36 (53.8)	
Heterozygous mutant (CC16 38A/G)	27 (37.5)	25 (37.3)	.78
Homozygous mutant (CC16 38A/A)	9 (12.5)	6 (9.0)	
FeNO, ppb, median (IQR)	10.4 (7.7‐18.7)	9.9 (1.7‐13.9)	.24
FeNO > 30 ppb, N (%)	10 (13.9)	9 (13.4)	.94

Abbreviations: FeNO, fractional exhaled nitric oxide; IgE, immunoglobulin E; IQR, interquartile range; NALF, nasal lavage fluid; ppb, parts per billion.

a Numbers of boys and girls: 55 and 52 for NALF biomarkers; 71 and 66 for urine biomarkers.

The odds of elevated FeNO (>30 ppb) were calculated according to the CC16 genotypes while adjusting or stratifying for the other significant predictors identified by the multiple regression analysis. As shown in Table 3, when considering the entire population, children with the 38AA genotype were about 10 times more likely to have an elevated FeNO with a statistically significant trend across the three genotype groups. Of interest, these associations between FeNO and the CC16 38AA genotype were if anything strengthened by excluding children sensitized to any aeroallergen. The stratification by gender revealed that these associations were driven by girls, being stronger by excluding boys (*P*_trend_ = .0001) while losing their statistical significance without girls (*P*_trend_ = .71). This interaction with the female gender also emerged when running the logistic regression model with the gender and 38AA genotype interaction term (odds ratio [OR], 15.8; 95% CI, 2.72 to 127, *P* = .01). When considering only girls or non‐sensitized children, the prevalences of elevated FeNO were also increased in subjects with the heterozygote 38AG genotype but without reaching the level of statistical significance.

**Table 3:** Odds ratios (OR) (with 95% confidence interval [CI]) of elevated FeNO according to CC16 single‐nucleotide polymorphism (SNP) genotype in all children or in children stratified according to aeroallergen sensitization or gender

		OR (95% CI)			
	n/N (%)	Unadjusted	Adjusted^a^	*P*	*P*_trend_
All children					
CC16 38G/G	6/71 (8.5)	1.0 (1.0‐1.0)	1.0 (1.0‐1.0)		.0045
CC16 38A/G	7/53 (13.2)	1.71 (0.54‐5.43)	2.26 (0.62‐8.30)	.22	
CC16 38A/A	6/15 (40.0)	7.33 (1.94‐27.7)	9.85 (2.09‐46.4)	.004	
Children sensitized to any aeroallergen					
CC16 38G/G	5/28 (17.9)	1.0 (1.0‐1.0)	1.0 (1.0‐1.0)		.044
CC16 38A/G	2/13 (15.4)	0.84 (0.14‐5.01)	1.11 (0.16‐7.62)	.91	
CC16 38A/A	4/6 (66.6)	9.20 (1.30‐64.9)	11.7 (1.44‐94.0)	.02	
Children not sensitized to any aeroallergen					
CC16 38G/G	1/43 (2.3)	1.0 (1.0‐1.0)	1.0 (1.0‐1.0)		.024
CC16 38A/G	5/38 (12.8)	6.36 (0.71‐57.1)	9.48 (0.74‐121)	.08	
CC16 38A/A	2/9 (22.2)	12.0 (0.96‐151)	40.4 (1.71‐951)	.02	
Girls					
CC16 38G/G	1/36 (2.8)	1.0 (1.0‐1.0)	1.0 (1.0‐1.0)		.0001
CC16 38A/G	4/25 (16.0)	6.67 (0.70‐73.7)	8.97 (0.84‐95.6)	.07	
CC16 38A/A	4/6 (66.7)	70.0 (5.13‐956)	107 (5.92‐1940)	.002	
Boys					
CC16 38G/G	5/36 (13.9)	1.0 (1.0‐1.0)	1.0 (1.0‐1.0)		.71
CC16 38A/G	3/27 (11.1)	0.78 (0.17‐3.57)	0.65 (0.10‐4.25)	.66	
CC16 38A/A	2/9 (22.2)	1.77 (0.28‐11.1)	2.17 (0.26‐17.8)	.40	

Abbreviations: FeNO, fractional exhaled nitric oxide; n, number of children with elevated FeNO according to the specified CC16 38A/G SNP; N, number of children with the specified CC16 38A/G.

a Adjusted for cleaning with bleach and for sensitization to any aeroallergen.

## 4 DISCUSSION

To our knowledge, our study is the first to report a positive association between the CC16 A38G polymorphism and the levels of FeNO. Children with the 38AA genotype had up to a 10‐fold increased odds of elevated FeNO compared with their peers with the 38GG genotype. This association emerged mainly in girls and was independent of the atopic status. Among girls and among non‐sensitized subjects, there was a borderline significant trend for an increase prevalence of elevated FeNO among children with the heterozygote 38AG genotype.

The CC16 A38G polymorphism is the main genetic determinant of serum CC16, a biomarker reflecting the amount of CC16 secreted in the deep lung. As previously reported, children with the mutant 38AA genotype show reduced plasma levels compared with the 38GG genotype.^5^ In light of the anti‐inflammatory properties of CC16, the association between FeNO and the CC16 A38G polymorphism, as found in this study, is most probably due to the lower intrapulmonary pool of this secretory protein in subjects with 38AA genotype. Of interest also, this association is characterized by a strong female gender specificity. Female‐dependent associations with genetic polymorphisms are not uncommon and have been reported for a variety of health conditions or diseases including asthma.^24^, ^25^ A modifying effect of gender was however, not described in studies that have explored the association between asthma and the A38G polymorphism. It is unclear whether this is because these studies found no influence of gender or simply because they did not investigate this issue. Further studies are needed to investigate in more depth the reason for this female gender‐specific association of elevated FeNO with the CC16 A38G genotype. Of note also, the association between the CC16 A38G polymorphism and FeNO was independent of atopy, suggesting that the CC16 38A allele predisposes to an increase of FeNO independently of the Th1/Th2 processes. A similar observation was made by Candelaria et al,^26^ who found a minimal influence of atopic status on the increased risk of asthma in adults with the 38AA genotype.

Several studies among children have shown that a high FeNO is predictive of developing asthma and impaired lung function later in life.^8^ The predictive value of an elevated FeNO level was established using FeNO cut‐off values between 22 and 35 ppb, which are rather comparable to that used in our study.^27^, ^28^ FeNO predicts asthma risk mostly in subjects with aeroallergen‐specific IgE (especially HDM), which, as also observed in our study, is strongly associated with elevated FeNO levels. Data are lacking regarding the predictive value of increased FeNO in non‐atopic children, who according to our study, are the most concerned by the association between FeNO and the CC16 A38G polymorphism. The study by Caudri et al^17^ showed that in schoolchildren, FeNO predicts asthma independently of IgE levels when combined with other risk factors such as maternal asthma or eczema. The increased odds of FeNO associated with the 38AA genotype independently of atopy might similarly be predictive of asthma risk whether or not associated with atopy.

The originality of our study in schoolchildren is that it relied entirely on noninvasive biomarkers. Urinary DNA was used for the CC16 SNP A38G genotyping assay, and has recently been shown to be a reliable biofluid, if saliva is not available.^23^ The observed allele frequencies followed the same trend as frequencies described by the 1000 Genome Project (45% 38GG, 44% 38AG, 11% 38AA for the CC16 A38G SNP).^29^ The slight deviation can be due to the relatively small sample size of our study and presumably also to ethnicity differences between our population and that of the 1000 Genome Project. Because blood samples were not available for measuring serum CC16, we used the concentrations of CC16 in urine or NALF as surrogate markers. The lack of association between CC16 levels in NALF and CC16 A38G is most probably due to the difficulty of properly adjusting NALF levels of CC16 for the variable recovery of CC16 by NALF and the physiological factors influencing the building up of the protein in the nasal ELF.^13^ The proper adjustment of the urinary CC16 levels was investigated with RBP4, a protein with similar biochemical properties and size to CC16, which has been described to be a classical protein candidate for tubular function.^15^, ^16^ Interestingly, urinary CC16 adjusted for RBP4 and chlorinated pool attendance was significantly reduced in children with 38AA as compared with those with the wild‐type 38GG. This decrease of urinary CC16 most likely mirrors the decrease of serum CC16 reported by Laing et al^10^ in subjects with the 38GG genotype.

The strong association of bleach house cleaning with FeNO is a rather unexpected finding as previous studies among domestic cleaners using bleach or among adolescents consistently reported no association between FeNO and the use of bleach.^30^, ^31^ This inconsistency might perhaps be explained by the younger age of our children and thus their greater sensitivity to the irritating effects of chlorine released from bleach.

Our study presents several limitations linked to the young age of children, which reduced the participation rate and success rate of the tests performed. The first limitation is the low numbers of children especially when stratifying the groups of genotypes according to gender or the atopic status. Fortunately, this limitation was balanced out by the particular strong statistical associations that were unlikely to be explained by chance only. Moreover, similar studies involving the investigation of for instance other genetic determinants and FeNO or sensitization parameters in small subject groups were also able to demonstrate strong associations.^13^, ^32^, ^33^ Nevertheless, studies with a higher number of subjects would be of interest to confirm the results of our study. Secondly, for ethical reasons we could not collect blood samples and check whether children with the 38AA genotype had lower serum levels of CC16 as reported previously. Thirdly, the lack of quantitative data on the individual exposure of children to air pollutants and other stressors may affect the levels of CC16 or exhaled NO and therefore weaken the associations of these biomarkers with CC16 A38G genotypes.

In conclusion, our findings suggest that the CC16 38AA allele promotes airway inflammation as measured by FeNO through a gender‐dependent association. Deficient levels of CC16 in the deep lung as reflected by lower CC16 urinary levels might underlie this promoting effect.

## CONFLICT OF INTERESTS

The authors declare that there are no conflict of interests.

## ETHICS STATEMENT

The Ethics Committee of the Faculty of Medicine of the Catholic University of UCLouvain approved the study protocol, which complies with all international regulations and applicable requirements. All human subjects participating in the study have given the requisite informed consent. More details can be found elsewhere.^18^

## Data Availability

All data that support the findings of this study are available in the tables of the manuscript. The individual data used to create these summarizing tables are available from the corresponding author upon reasonable request.

## References

[1] Broeckaert F , Bernard A. Clara cell secretory protein (CC16): characteristics and perspectives as lung peripheral biomarker. Clin Exp Allergy. 2000;30:469‐475. 10718843 10.1046/j.1365-2222.2000.00760.x

[2] Hermans C , Bernard A. Lung epithelium‐specific proteins: characteristics and potential applications as markers. Am J Respir Crit Care Med. 1999;159(2):646‐678. 10.1164/ajrccm.159.2.9806064 9927386

[3] Hung C‐H , Chen L‐C , Zhang Z , et al. Regulation of TH2 responses by the pulmonary Clara cell secretory 10‐kd protein. J Allergy Clin Immunol. 2004;114(3):664‐670. 10.1016/j.jaci.2004.05.042 15356574

[4] Mango GW , Johnston CJ , Reynolds SD , Finkelstein JN , Plopper CG , Stripp BR . Clara cell secretory protein deficiency increases oxidant stress response in conducting airways. Am J Physiol. 1998;275(2):L348‐L356. 10.1152/ajplung.1998.275.2.L348 9700096

[5] Shijubo N , Itoh Y , Yamaguchi T , et al. Serum levels of Clara cell 10‐kDa protein are decreased in patients with asthma. Lung. 1999;177(1):45‐52. 10.1007/PL00007626 9835633

[6] Lomas DA , Silverman EK , Edwards LD , et al. Evaluation of serum CC‐16 as a biomarker for COPD in the ECLIPSE cohort. Thorax. 2008;63(12):1058‐1063. 10.1136/thx.2008.102574 18757456

[7] Bernard A , Nickmilder M , Dumont X. Chlorinated pool attendance, airway epithelium defects and the risks of allergic diseases in adolescents: interrelationships revealed by circulating biomarkers. Environ Res. 2015;140:119‐126. 25863185 10.1016/j.envres.2015.03.034

[8] Pijnenburg MW . The role of FeNO in predicting asthma. Front Pediatr. 2019;7:41 10.3389/fped.2019.00041 30847334 PMC6393362

[9] Broeckaert F , Arsalane K , Hermans C , et al. Serum Clara cell protein: a sensitive biomarker of increased lung epithelium permeability caused by ambient ozone. Environ Health Perspect. 2000;108(6):533‐537. 10856027 10.1289/ehp.00108533 PMC1638141

[10] Laing IA , Hermans C , Bernard A , Burton PR , Goldblatt J , Le Souëf PN . Association between plasma CC16 levels, the A38G polymorphism, and asthma. Am J Respir Crit Care Med. 2000;161(1):124‐127. 10.1164/ajrccm.161.1.9904073 10619808

[11] Taniguchi N , Konno S , Hattori T , et al. The CC16 A38G polymorphism is associated with asymptomatic airway hyper‐responsiveness and development of late‐onset asthma. Ann Allergy Asthma Immunol. 2013;111(5):376‐381. 10.1016/j.anai.2013.08.005 e1. 24125144

[12] Cheng D , Di H , Xue Z , Zhen G. CC16 gene A38G polymorphism and susceptibility to asthma: an updated meta‐analysis. Intern Med. 2015;54(2):155‐162. 10.2169/internalmedicine.54.2979 25743006

[13] Bernard A , Sardella A , Voisin C , Dumont X. Nasal epithelium injury by chlorination products and other stressors predicts persistent sensitization to aeroallergens in young schoolchildren. Environ Res. 2017;158:145‐152. 10.1016/j.envres.2017.06.009 28628840

[14] Wang H , Dumont X , Haufroid V , Bernard A. The physiological determinants of low‐level urine cadmium: an assessment in a cross‐sectional study among schoolchildren. Environ Health. 2017;16(1):99 10.1186/s12940-017-0306-5 28899425 PMC5596934

[15] Norden AGW , Lapsley M , Unwin RJ . Urine retinol‐binding protein 4: a functional biomarker of the proximal renal tubule. Adv Clin Chem. 2014;63:85‐122. 10.1016/B978-0-12-800094-6.00003-0 24783352

[16] Weise M , Prüfer D , Jaques G , Keller M , Mondorf AW . β‐2‐microglobulin and other proteins as parameter for tubular function. Contrib Nephrol. 1981;24:88‐98. 10.1159/000395233 6164517

[17] Caudri D , Wijga AH , Hoekstra MO , et al. Prediction of asthma in symptomatic preschool children using exhaled nitric oxide, Rint and specific IgE. Thorax. 2010;65(9):801‐807. 10.1136/thx.2009.126912. 20805175

[18] Voisin C , Sardella A , Bernard A. Risks of new‐onset allergic sensitization and airway inflammation after early age swimming in chlorinated pools. Int J Hyg Environ Health. 2014;217(1):38‐45. 10.1016/j.ijheh.2013.03.004 23601779

[19] Marcucci F , Passalacqua G , Canonica GW , et al. Measurement of nasal IgE in an epidemiological study: assessment of its diagnostic value in respiratory allergy. Eur Ann Allergy Clin Immunol. 2004;36(6):225‐231. 15329005

[20] Marcucci F , Sensi L. A new method for IgE detection in nasal mucosa. Clin Exp Allergy. 1989;19(2):157‐162. 10.1111/j.1365-2222.1989.tb02358.x 2752318

[21] Bernard A , Lauwerys RR . Continuous‐Flow system for automation of latex immunoassay by particle counting. Clin Chem. 1983;29(6):1007‐1011. 6342848

[22] Bernard A , Roels H , Lauwerys R , et al. Human urinary protein 1: evidence for identity with the Clara cell protein and occurrence in respiratory tract and urogenital secretions. Clin Chim Acta. 1992;15(207):239‐249. 10.1016/0009-8981(92)90122-7 1395029

[23] Nauwelaerts SJD , Van Geel D , Delvoye M , et al. Selection of a noninvasive source of human DNA envisaging genotyping assays in epidemiological studies: urine or saliva? J Biomol Tech. 2020;31(1):27‐35. 10.7171/jbt.20-3101-004 jbt.20‐3101‐004. 32042275 PMC6977458

[24] Wu C‐C , Ou C‐Y , Chang J‐C , et al. Gender‐dependent effect of GSTM1 genotype on childhood asthma associated with prenatal tobacco smoke exposure. BioMed Res Int. 2014;2014:769452 10.1155/2014/769452 25328891 PMC4189933

[25] Jiménez‐Morales S , Martínez‐Aguilar N , Gamboa‐Becerra R , et al. Polymorphisms in metalloproteinase‐9 are associated with the risk for asthma in Mexican pediatric patients. Hum Immunol. 2013;74(8):998‐1002. 10.1016/j.humimm.2013.04.036 23639553

[26] Candelaria PV , Backer V , Laing IA , et al. Association between asthma‐related phenotypes and the CC16 A38G polymorphism in an unselected population of young adult Danes. Immunogenetics. 2005;57(1):25‐32. 10.1007/s00251-005-0778-2 15744536

[27] Di Cara G , Marcucci F , Palomba A , et al. Exhaled nitric oxide in children with allergic rhinitis: a potential biomarker of asthma development. Pediatr Allergy Immunol. 2015;26(1):85‐87. 10.1111/pai.12326 25511873

[28] Woo S‐I , Lee J‐H , Kim H , Kang J‐W , Sun Y‐H , Hahn Y‐S . Utility of fractional exhaled nitric oxide (FENO) measurements in diagnosing asthma. Respir Med. 2012;106(8):1103‐1109. 10.1016/j.rmed.2012.03.022 22534041

[29] The 1000 Genomes Project Consortium . A map of human genome variation from population‐scale sequencing. Nature. 2010;467(7319):1061‐1073. 10.1038/nature09534 20981092 PMC3042601

[30] Nickmilder M , Carbonnelle S , Bernard A. House cleaning with chlorine bleach and the risks of allergic and respiratory diseases in children. Pediatr Allergy Immunol. 2007;18(1):27‐35. 10.1111/j.1399-3038.2006.00487.x 17295796

[31] Sastre J , Madero MF , Fernández‐Nieto M , et al. Airway response to chlorine inhalation (bleach) among cleaning workers with and without bronchial hyperresponsiveness. Am J Ind Med. 2011;54(4):293‐299. 10.1002/ajim.20912 20957677

[32] Saadat M , Saadat I , Saboori Z , Emad A. Combination of CC16, GSTM1, and GSTT1 genetic polymorphisms is associated with asthma. J Allergy Clin Immunol. 2004;113(5):996‐998. 10.1016/j.jaci.2004.02.007 15148962

[33] Carbonnelle S , Bernard A , Doyle IR , Grutters J , Francaux M. Fractional exhaled NO and serum pneumoproteins after swimming in a chlorinated pool. Med Sci Sports Exerc. 2008;40(8):1472‐1476. 10.1249/MSS.0b013e3181733159 18614944

